# Mechanisms and Potential Clinical Implications of Oral Microbiome in Oral Squamous Cell Carcinoma

**DOI:** 10.3390/curroncol31010011

**Published:** 2023-12-28

**Authors:** Jingyi Wang, Bo Gao

**Affiliations:** State Key Laboratory of Oral Diseases, National Clinical Research Center for Oral Diseases, West China Hospital of Stomatology, Sichuan University, Chengdu 610041, China; wangjingyi3@stu.scu.edu.cn

**Keywords:** oral squamous cell carcinoma, oral potentially malignant disorders, oral microbiome, inflammation, immunity

## Abstract

Microorganisms in the oral cavity are abundant in the human body. At present, more than 700 species of oral microorganisms have been identified. Recently, a lot of literature has indicated that the oral microbiota plays an important role in the occurrence, development, and prognosis of oral squamous cell carcinoma (OSCC) through various mechanisms. And researchers are now trying to utilize oral microbiota in cancer diagnosis and treatment. However, few articles systematically summarize the effects of oral microbes in the diagnosis, treatment, and disease outcomes of oral cancer. Herein, we made a summary of the microbial changes at cancerous sites and placed more emphasis on the mechanisms by which the oral microbiome promotes cancerization. Moreover, we aimed to find out the clinical value of the oral microbiome in OSCC.

## 1. Introduction

More than 90% of oral cancers, which rank 16th among all the common malignant tumors, are oral squamous cell carcinomas (OSCCs) originating from the squamous tissues [1]. Advances in medical imaging and therapy have improved the 5-year overall survival from 59% during 1990–2000 to 70% during 2001–2010 [2]; however, there were 377,713 cases of oral and lip cancer and 177,757 deaths in 2020 [3]. The etiology of OSCC is attributed to genetics; microbes; and unhealthy habits, including alcoholism [4], smoking [5], and chewing betel [6]. Periodontal diseases and tooth loss are also risk factors for OSCC [7,8], indicating that several oral bacteria (*Streptococcus*, *Peptostreptococcus*, and *Prevotella*) may be related to the development of OSCC [9]. Microorganisms that colonize the human body were found to be associated with 20% of cancers and are known to modulate tumor occurrence and development since *Helicobacter pylori* (*H. Pylori*) was found to contribute to gastric cancer in the 1990s [10]. The number of bacteria is almost equal to that of human cells in the body, and these microorganisms have at least 100 times more metagenomes than humans, which can be utilized to modulate the biological behavior of cancer cells [11] by promoting cell proliferation, resisting cell death, inducing angiogenesis, reprogramming energy metabolism, and evading immune destruction [12]. Increasing evidence suggests that microorganisms play a significant role in oral cancer development. Therefore, the mechanisms of microbial carcinogenesis should be explored, and a cure must be developed for oral cancer. In this review, we summarized the current knowledge of oral microbiota and its relationship with OSCC, placed an emphasis on the mechanisms by which oral microbiota promote oral cancer, and aimed to determine the potential clinical role of oral microbiota in OSCC.

## 2. Oral Microbiota Participating in the Occurrence, Development, and Prognosis of OSCC

### 2.1. Oral Microbiota and Oral Potentially Malignant Disorders 

Transformation of normal oral mucosa into OSCC involves multiple steps, of which oral potentially malignant disorders (OPMDs) have received increasing attention owing to the high risk of malignant transformation (the overall malignant conversion rate is 7.9% [13]). More than 80% of oral cancer cases develop from OPMDs [14]. Common OPMDs with a high risk of transformation include oral leukoplakia, proliferative verrucous leukoplakia, erythroplakia, oral lichen planus (OLP), and oral submucous fibrosis. 

Although the microbiome community structure in oral cancer is different from that in normal samples, the community in OPMDs overlaps with both [15]. Specifically, according to the research of Amer et al., 35% of leukoplakia samples showed considerable *Candida* spp. colonization (>300 CFUs/mL) [16]. *Candida* spp. produce carcinogens and proteinases that degrade the basement membrane [17]. However, patients with leukoplakia have high levels of *Fusobacteria* and *Bacteriodetes* and low levels of *Firmicutes* [16,18]. Interestingly, *Firmicutes* were often identified in the cancer group. Additionally, many periodontal pathogens (*F. nucleatum*, *P. intermedia*, and *P. gingivalis*) are found in patients with both oral leukoplakia (*OLK*) and oral cancer. Although similar bacteriomes were detected in the whole-mouth fluid of patients with oral leukoplakia and OSCC, there were slight differences in *Megasphaera*, *unclassified Enterobacteriae*, *Salmonella*, and *Prevotella*. For instance, *Rothia mucaliginosa* is abundant in OLK lesions, but less abundant in OSCC [19]. Thus, it can be inferred that some oral bacteria may be driving factors in the early stages of oral carcinogenesis. *C. albicans* was found to shield *P. gingivalis* by decreasing cytokine and chemokine production and reducing macrophage and fibroblast responses [20], indicating a protective effect among oral microbes. So far, viruses in OPMDS have rarely been reported. A meta-analysis found that HPV 16/18 was strongly associated with OLP and OLK [21]. In general, continuous microbial changes are observed throughout the development of OSCC [22]. However, further research is required to elucidate the relationship between microbes and the risk of malignant transformation [23]. 

### 2.2. Oral Microbiota and OSCC

Yang et al. identified *Fusobacterium periodonticum*, *Parvimonas micra*, *Streptococcus constellatus*, *Haemophilus influenza*, and *Filifactor alocis* as the five most representative microorganisms in patients with OSCC [24]. Periodontitis-related bacteria (including *Prevotella tannerae*, *F. nucleatum*, and *P. intermedia)* associated with dentition loss and poor oral condition were considered risk factors for OSCC [7,8,25,26]. However, Hayes et al. argued the opposite [27]. The HPV and Epstein–Barr virus (EBV) are two popular viruses associated with OSCC [28]. The number of patients with HPV-positive HNSCC has been increasing, especially those with pharyngeal cancer [29], and these patients present with a significantly better prognosis than patients with HPV-negative HNSCC under similar treatments. Therefore, HNSCC is further divided into HPV-positive and HPV-negative types, which is conducive to exploring different causes and treatment options. The EBV is related to OSCC as well, as 55% of 155 OSCC samples from eight different countries and ethnicities were EBV-positive [30,31]. As to yeasts, *Candida albicans* is more prevalent in patients with oral cancer than in healthy people [32]. 

**Figure 1:**
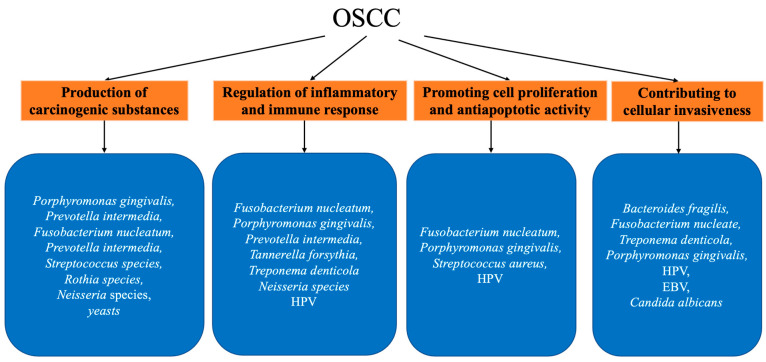
Summary of mentioned oral microbiota and their association with OSCC.

The mechanisms of oral microbial carcinogenesis have been extensively studied and can be roughly summarized as follows (Figure 1): (I) production of carcinogenic substances, (II) regulation of inflammatory and immune responses, (III) promotion of cell proliferation and anti-apoptotic activity, and (IV) contribution to cellular invasiveness (discussed in Section 2.3).

#### 2.2.1. Production of Carcinogenic Substances

Microorganisms produce metabolites, such as nitrosamines, sulfides, oxides, and acetaldehyde, which induce tumorigenesis. In advanced precancerous lesions, *Candida albicans* strains with high nitrosation potential can be isolated [33], whereas nitrosamines can interfere with DNA replication by binding to chemical bonds in the DNA, which contributes to carcinogenesis. Volatile sulfur compounds produced by *P. gingivalis*, *P. intermedia*, and *F. nucleatum* modulate cellular behavior [34]. Yaegaki et al. found that after incubation with hydrogen sulfide (H_2_S) for 72 h, histone-complexed DNA fragmentation in human gingival fibroblasts increased, resulting in apoptosis [35,36]. Moreover, reactive oxygen species (ROS) and reactive nitrogen species secreted by microbiota-induced inflammatory cells contribute to tumor invasion, angiogenesis, and metastasis. Another possible hypothesis is that bacteria contribute to the development of oral cancer by activating procarcinogens. *Streptococcus*, *Rothia*, *P. gingivalis*, *Neisseria*, yeasts, and fungi possess alcohol dehydrogenase (ADH), which is an enzyme that can metabolize ethanol to acetaldehyde (ACH) [37,38]. As shown by Alnuaimi et al., highly ethanol-derived ACH-producing *Candida* could be isolated from patients with oral cancer [39]. Although pure ethanol is not carcinogenic, ACH leads to the formation of DNA protein adducts, causing point mutations and chromosomal aberrations by intervening in normal DNA replication. Smedra proposed a different point of view that ACH can originate from oral autobrewery syndrome owing to microbial dysbiosis in the oral cavity, aside from the breakdown of ingested alcohol by oral mucosal ADH, the oral microbiome, and alcoholic beverage itself [40]. 

#### 2.2.2. Regulating Inflammatory and Immune Suppression

The link between cancer and inflammation has been recognized and confirmed by epidemiological investigations and experimental studies [41,42]. Toll-like receptors (TLRs) are host pattern recognition receptor molecules that recognize lipopolysaccharide (LPS), which is a component of the Gram-negative bacterial cell wall, bacterial flagellin, and bacterial nucleic acids. In response to LPS activation, inflammatory cells (such as monocytes/macrophages, neutrophils, fibroblasts, and mast cells) secrete IL-1β, which are in charge of the release of phospholipase A2, prostaglandins, acute phase proteins, proinflammatory cytokine IL-6, and TNF. On the one hand, IL-1β promotes angiogenesis and tumor progression by activating endothelial cells, which produce vascular endothelial growth factor and other proangiogenic factors (e.g., TNF). On the other hand, IL-1β enhances tumor invasiveness and aggression by decreasing E-cadherin expression and inducing MMP-9. IL-6 induces oxidative stress and causes mitochondrial damage. Moreover, TNF-α can produce ROS to induce DNA damage and contribute to tumor progression through angiogenesis. Through the TLR/MyD88 pathway, the transcription factor nuclear factor of kappa beta (NF-κB) is activated, contributing to inflammation and cell proliferation. Periodontitis-related bacteria also initiate the overexpression of NLRP3 and activate the upstream signaling molecules of ATR-CHK1 [43]. Chymotrypsin-like proteinase (CTLP), which is a major virulence factor of *T. denticola*, is critical for regulating inflammation by degrading complement C1q [44]. 

Microbiome-related immunosuppression plays a significant role in the development of oral cancer by modulating immune cell activity. *P. gingivalis* suppresses CD8+ T-cell cytotoxicity by upregulating PD-L1 expression in dendritic cells through Akt-STAT3 signaling [45]. By increasing the FOXP3 + Treg response, *P. gingivalis* induces monocytes to become myeloid-derived dendritic suppressor cells, resulting in immune tolerance [46]. Upregulation of the immune checkpoint molecule B7-H4 and lysine demethylation of 5 B induced by *P. gingivalis* also promotes evasion and inhibition of the host immune response [47]. Gingipain K produced by *P. gingivalis* degrades immunoglobulins and the complement system (C3 and C5 components) and affects the host immune system [48]. Gur et al. found that the Fap2 protein of *F. nucleatum* interacts with TIGIT, which is an inhibitory receptor on all human NK cells and various T cells and assists tumor cells in escaping immune cell attack [49]. In addition to promoting the M2 polarization of macrophages through the TLR4-associated pathway [50], *F. nucleatum* induces cancer cells to produce more lactate via the GalNAc–Autophagy–TBC1D5–GLUT1 signaling axis, subsequently promoting M2-like tumor-associated macrophage formation and contributing to immune evasion [51]. In addition to bacteria, viruses, such as HPV, can promote cancer development through sophisticated immunity escape techniques. HPV E7 oncogene has been reported to inhibit the activation of NF-κβ family members, leading to viral immunosuppression [52].

#### 2.2.3. Promoting Cell Proliferation and Antiapoptotic Activity

Oral microbes facilitate cancer development by promoting cell proliferation and anti-apoptotic activities. Cell cycle dysregulation is one of the most common features of human cancers. *P. gingivalis* contributes to OSCC proliferation by accelerating the G1 phase of the cell cycle through the upregulation of cyclin D1 expression via the miR-21/PDCD4/AP-1 signaling pathway [53]. The intact fimA fimbrial structure and gingipain proteases of *P. gingivalis* also assist in cancer cell apoptosis and proliferation [54,55]. In the presence of *F. nucleatum*, p27, which is associated with abnormal cell proliferation, is downregulated, causing cell cycle arrest in the S phase and strengthening cell proliferation [56]. Meanwhile, the DNA repair proteins Ku70 and p53 are downregulated, weakening their cell repair ability [56]. p53, along with pRb, is the target substrate of HPV E6/E7 oncogenes, leading to continual cell proliferation. *F. nucleatum* is also associated with high levels of putrescine, which induces the malignant proliferation of cancer cells [57]. *Staphylococcus aureus* can facilitate human oral keratinocyte proliferation by activating the COX-2/PGE2 pathway [58].

The body clears necrotic cells through apoptosis or programmed death, which may interfere with pathological conditions and lead to tumor formation. Apart from reducing IL-1β production and inhibiting ATP-dependent apoptosis, nucleoside-diphosphate-kinase secreted by *P. gingivalis* also abrogates epithelial cell death by phosphorylating heat-shock-protein-27 [59]. Through the JAK1/AKT/STAT3 pathway, *P. gingivalis* phosphorylates the pro-apoptotic protein Bad and inhibits the release of cytochrome c, reducing the intrinsic mitochondrial apoptotic activity [60,61]. Similarly, in a study of infection with *F. nucleatum*, the expression of MYC, JAK1, and STAT3 was significantly stimulated [62]. *T. denticola* can invade CAL-27 cells and directly promote cell proliferation, regulate the cell cycle, and inhibit cell apoptosis [63]. Recently, HPV E7 was found to inhibit pyroptosis by recruiting TRIM21 for ubiquitination and degradation of the IFI16 inflammasome [64]. 

### 2.3. Microbiome and Oral Cancer Prognosis

The oral microbiome also promotes cancer development by increasing cellular migration and invasiveness, leading to the aggressiveness and metastasis of OSCC, which is associated with a poor prognosis. 

The epithelial–mesenchymal transition (EMT) is a cell-reprogramming process by which cancer cells complete differentiation and become aggressive [65]. Upon infection by *P. gingivalis*, both the JAK1/STAT3 and EMT phenotypes are activated, and tumor-associated neutrophils are recruited to promote OSCC progression via the CXCL2/CXCR2 axis [66]. Tumor growth and lung metastasis are remarkably enhanced by activating TGFβ signaling. The *Bacteroides fragilis* toxin cleaves E-cadherin, resulting in a decrease in cell–cell attachment. *Fusobacterium* adhesin A (FadA) also dissociates adherence junctions by disconnecting the E-cadherin–β-catenin complex [67]. β-catenin then transits to the nucleus and induces the Wnt pathway. Wnt target genes, including ZEB1, SNAI1, MT1-MMP-9, and LAMC2, are also associated with invasiveness. Although some studies declared that *Fusobacterium* is related to a reduced recurrence rate and a low rate of lymph node metastasis [68,69], others found that *Fusobacteria* can enhance cancer cell invasiveness, survival, and EMT via the JAK/STAT3 pathway [62] and lncRNA MIR4435-2HG/miR-296-5p/Akt2/SNAI1 signaling pathway [70]. Matrix metalloproteinases (MMPs) degrade extracellular matrix components, enabling tumor cells to spread [71]. *P. gingivalis* increases signaling through extracellular signal-regulated kinase 1/2-Ets1, p38/HSP 27, and NF-κB pathways, consequently elevating MMP-9 and downregulating E-cadherin [72]. MMPs can also be activated by methyl mercaptan by increasing the production of IL-1 and prostaglandin E2, thereby facilitating the degradation of collagen, especially type 4 collagen, and reducing its synthesis, which results in the inhibition of basement membrane synthesis [35]. EBV inhibits P53 via the CTAR family of proteins/programmed cell death protein 1 ligands to enhance the migration of epithelial cells and the production of MMPs [73]. In addition, Vadovics et al. demonstrated that *C. albicans* infection, instead of zymosan treatment, significantly increases the total MMP activity by means of *C. albicans* hypha generation [20], which can be further explained by Ho’s study, which showed that the hypha-specific toxin candidalysin induces MMP activation and expression via a calcium influx [74]. Similarly, CTLP was found to increase cellular migration and tumor invasion by upregulating MMP-2 via TLR-7 and TLR-9 [75], as well as TLR/MyD88 and integrin/FAK crosstalk signaling [76]. Cytoskeleton remodeling is attributed to the EMT, which enhances cell mobility. Cytoskeleton assembly is realized by HPV16 E6 by downregulating NHERF1 to promote cell invasiveness [77].

## 3. Clinical Application of Oral Microbes in OSCC

### 3.1. Diagnosis and Grading

Several advanced approaches can detect oral cancer, such as lab-on-chip, microfluidics, nanodiagnostics, liquid biopsy, omics technology, and synthetic biology [78]. To date, numerous studies have applied different methods to describe the differences in oral microbiota between normal tissue and OSCC sites [28,79,80,81], including the surface of the tumor tissue, within the tumor, and saliva. Allan Radaic etc. made an exhaustive summary of potential oral microbiome-based biomarkers for OSCC [82]. It is possible to distinguish cancerous lesions from normal tissues and perform tumor staging by noninvasively detecting microbes [83]. 

Intratumoral bacteria potentially originate from normal adjacent tissues [84] and play the role of immunomodulation in the tumor microenvironment [85]. Hooper et al. were the first to study microorganisms in OSCC and suggested that they are mainly aciduric and saccharolytic secondary colonizers, such as *Micrococcus luteus*, *Prevotella melaninogenica*, *Exiguobacterium oxidotolerans*, and *Staphylococcus aureus*, because of their acidic and hypoxic environments [86]. The abundances of the phylum *Fusobacteria*, genus *Fusobacterium*, and phylum *Bacteroidetes* were found to be elevated in the saliva of patients with OSCC and were believed to be diagnostically specific [15]. Aside from viruses and bacteria, a variety of molecular and genetic markers can be detected for treatment and monitoring [87]. A recent study reported that oral-cancer-related microorganisms in the mucosa, other than in gingival plaque or saliva samples, have the most diverse species differences and functional changes and are the most suitable sites for observing microbial dysregulation [88]. Another study that investigated unstimulated salivary microbial profiles found significant differences in *Bacillus*, *Enterococcus*, *Parvimonas*, *Peptostreptococcus*, and *Slackia* between epithelial precursor lesions and cancer groups [89]. As early as 2005, Madhura et al. found that with *Capnocytophaga gingivalis*, *Prevotella melaninogenica*, and *Streptococcus mitis* as diagnostic markers, the sensitivity and specificity for the three species are 80% and 82%, respectively [90]. Zhou et al. adopted random forests and cross-validations to build a diagnostic model based on oral microbiota and found that Actinobacteria, Fusobacterium, Moraxella, Bacillus, and Veillonella species were strongly correlated with OSCC [91]. However, *Parvimonas micra* and *Streptococcus mitis* have been implicated in the reduced risk of OSCC development [27,92,93,94]. The presence of *Corynebacterium* and *Kingella* is also associated with a low incidence of head and neck squamous cell carcinoma, possibly because they are involved in the degradation of cancer-inducing metabolites [27,95]. Similarly, Shen et al. found that periodontitis-negative-associated bacteria (*Neisseria sicca* and *Corynebacterium matruchotii)* play an anti-cancer role in OSCC by upregulating DDR to repair DNA damage, inducing pyroptosis, and decreasing CD4+ T cells [96]. *P. gingivalis* IgG and IL-6 are also used as potential serum biomarkers for OSCC diagnosis [97]. As the expression patterns of CXCL10, DIAPH1, NCLN, and MMP9 genes are significantly correlated with interpain A, fadA, and bspA in OSCC cases, gene expression is an alternative target to detect OSCC [98]. 

With the progression of OSCC, the abundance of these bacteria increases, indicating that the microbiome can serve as a marker for staging and predicting prognosis. Yang et al. reported that *F. periodonticum*, *S. mitis*, and *P. pasteri* are bacterial marker panels that can be used to distinguish patients with stage 4 OSCC from healthy individuals [24]. Tumor MMP-9 expression is associated with poor outcomes in OPSCC, especially in HPV-negative disease, whereas Rgp immunoexpression in inflammatory cells is associated with better disease-specific survival, which can be utilized to predict prognosis [99].

In addition to diagnosing the disease, microorganisms can also be used to distinguish healthy from diseased mucosa. Su et al. demonstrated that *Fusobacterium* spp. is a successful marker species for identifying noncancerous tissues. However, compared with *Fusobacterium* spp., *Streptococcus* spp., especially *Streptococcus pneumoniae*, are more accurate when classifying lesion sites [80].

### 3.2. Oral Microbes and Cancer Treatment 

Multidisciplinary therapeutic strategies are generally used for OPMDs to prevent OSCC progression and prolong survival. However, there is no consensus on the treatment of OPMDs owing to the variety and complex mechanisms of OPMDs. Tacrolimus, which is used as the first-line drug after transplantation, was found to be effective in treating OLP [100,101] by downregulating immunity [102] and downregulating cell-cycle-related proteins [103]. Tacrolimus treatment also significantly altered the proportion of *Allobaculum*, *Bacteroides*, and *Lactobacillus* in the colonic mucosa and the circulation [104], which indicated that it may improve microbial dysregulation of mucosal surfaces. Some studies reported that tacrolimus promotes tumorigenesis and leads to adverse events [105,106], while others have reached a different conclusion [107,108,109]. Topical tacrolimus can be an effective second-line therapy for patients who do not respond to corticosteroids. However, further studies on its adverse effects are required.

A combination of surgery, chemotherapy, and radiotherapy has been administered to patients with OSCC. Recently, more attention has been paid to the oral microbiome, as the structural, metabolic, and virulence characteristics of microbes are potential targets. The use of pre- or probiotics and salivary substitutes was also suggested based on differences in the salivary microbiota between patients with OSCC and healthy controls [110,111]. The concept of oncolytic bacterial immunotherapy has long been popular, as commonly used radiotherapy and chemotherapy have side effects owing to damage to healthy tissues, while microorganism therapy shows the merits of accurate target specificity, tissue penetration, and less treatment expense. Many engineered bacterial strains were generated to overcome potential safety problems and improve tumor targeting [112]. *Salmonella typhimurium* [113], *Escherichia coli* [114], and *Bifidobacterium* [115] showed outstanding anti-cancer activities in both preclinical and clinical trials. *Bifidobacterium*, *Streptococcus*, *Caulobacter*, and *Clostridium* spp. are commonly found in the oral cavity and are promising candidates for OSCC tumor-targeting therapies [116]. Oncolytic or “cancer-killing” viruses have been highly used as immunotherapeutic drugs for the treatment of cancer as well [117] and are combined with radiotherapy, chemotherapy drugs, or other strategies. Nonetheless, concerns remain regarding the use of microorganisms in tumor therapies. Owing to the weakened immune system, bacteria-mediated tumor therapy is ineffective in patients who have undergone chemotherapy [118] and can cause serious infections. Moreover, bacterial monotherapy does not completely cure cancer, and bacterium-mediated synergistic cancer therapy was proposed to have promising potential [119,120]. Further studies are warranted to understand the interactions between oncolytic immunotherapies and other therapies.

### 3.3. Microbiota and Treatment Outcomes of OSCC

Increasing evidence indicates that the presence of a microbiome can affect treatment outcomes [121]. Recent research found an increase in *Lactobacillaceae* and *Bifidobacteriaceae* families and a decrease in *Porphyromonadaceae* and *Prevotellaceae* after OSCC treatment. Furthermore, they observed a change in DMBT1 expression accompanied by the microbiome change, suggesting DMBT1 to be a possible treatment indicator [122]. And a certain genus, namely, *Leptotrichia*, was shown to improve patient prognosis [123]. Another prediction model with five microbial signatures, namely, *Leptotrichia trevisanii*, *Capnocytophaga sputigena*, *Capnocytophaga*, *Cardiobacterium*, and *Olsenella*, displayed high accuracy [124]. Cancer-related intratumoral bacteria and gut microbiota influence the effectiveness of chemotherapy. Lehouritis et al. found that bacteria can both decrease or increase the effectiveness of chemotherapeutic drugs via enzymatic biotransformation and chemical modification [125]. In a mouse model of colon cancer, intratumor Gammaproteobacteria converted gemcitabine into its inactive form by expressing the bacterial enzyme cytidine deaminase [126]. Another study found that 5-FU is metabolized by *preTA*-encoding bacteria [127]. Microbes modulate drug toxicity and side effects. In mice raised in a germ-free environment and administered antibiotic prophylaxis, oxyaliplatin-induced mechanical hyperalgesia was reduced, indicating that the gut microbiota enhanced chemotherapy-induced mechanical hyperalgesia [128]. Simultaneously, because microorganisms have a regulatory effect on immune-inflammatory responses, they also affect immunotherapy. *Fusobacteria* species are associated with the high expression of IL-12 and TGF-β, ultimately promoting the differentiation of T cells [129]. As for radiotherapy, antibiotic-mediated fungal reduction enhances the response to radiation, whereas antibiotic-mediated bacterial reduction presents the opposite results [130]. Significant microbiome changes can also affect radiation-induced osteoradionecrosis [131]. Current research on the microbial effects of cancer therapy mainly focuses on digestive tract cancers, requiring further exploration for oral cancer. Although the mechanism by which these microbes affect the efficacy of anti-cancer treatments remains unclear, a growing number of studies have shown that microbes are inextricably linked to anti-cancer treatments.

In addition to regulating cancer treatment, the microbiome can also be a target for regulating immune-related adverse events and even the prognosis of cancer treatment, as cancer treatments can lead to microflora dysregulation. Almost 75% of patients with head and neck cancer who undergo chemotherapy or radiotherapy treatment experience oral mucositis (OM) [132]. After receiving a 5-fluorouracil (5-FU) i.v. for 6 d, an increase in facultative and strictly anaerobic bacteria in the oral cavity and facultative anaerobes in the colon is observed [133]. Hong et al. also demonstrated that OM severity is associated with 5-FU [134]. Modification of oral microbiome is a promising preventive treatment for OM [135,136]. Palifermin, which is the only pharmacological agent approved by the FDA to treat OM, is a recombinant human keratinocyte growth factor (KGF) that targets the KGF receptor to enhance the differentiation and maturation of epithelial cells [132,137]. A recent meta-analysis showed that palifermin reduces the incidence of severe mucositis by up to 30% in patients treated with chemotherapy and radiotherapy [138]. Therefore, palifermin can be used as a prophylactic to prevent severe OM in patients with oral cancer. Recent research found that palifermin affects the oral microbial community composition, though more studies are warranted to figure out the correlations between palifermin and community composition changes [139]. However, in the 2021 European clinical practice guidelines, palifermin is not recommended for pediatric patients receiving cancer treatment [140] because of its short-term adverse effects, potential long-term negative effects on cancer outcomes, high costs, and restricted availability. Palifermin can be used as a prophylactic to prevent severe OM in patients with oral cancer; more reliable evidence is needed on the safety and efficacy of palifermin. Interestingly, *Lactococcus* strains were shown to be effective in the control of 5-FU-dysbiosis [141], indicating that probiotic supplementation may be a prophylaxis to reduce the adverse effects of cancer therapies and improve the quality of patients’ lives. Table 1 summarizes the promising clinical applications of oral microbes in OSCC.

## 4. Conclusions

This review specifically introduces the mechanism of oral microbiota in OSCC. The oral microbiome can facilitate the occurrence and development of OSCC by producing carcinogens, promoting cell proliferation, suppressing cell death, and finally inducing angiogenesis. They can also impact the prognosis by enhancing invasiveness and metastasis. To sum up, strong evidence demonstrates that certain genera and species do play a specific role in the development of OSCC, while research has yet to clearly figure out the mechanisms by which oral microbiota lead to tumorigenesis. In addition, using oral microbes for treatment presented promising prospects given the fact that oral microbes have an impact on the therapeutic effect.

Although previous studies demonstrated changes in the oral microbiota during the development of OSCC and identified several typical pathogens, contradictory statements have been reported in the literature on some species, which were probably caused by different detection methodologies and different detection sites. 16S rRNA sequencing is the most popular technology for detecting the oral microbiome; however, it may be restricted by its small sample size [28]. Therefore, other molecular techniques for quantitative determination, such as LDA effect size analysis [68], fluorescent quantitative PCR, and flow cytometry [142], were introduced to obtain more accurate results. Additionally, many factors (habit, race, region, and economic background) can influence the composition of the oral microbiome, which should be excluded during sample collection.

Moreover, the progression of OSCC is complex. The inflammatory state caused by oral microbial dysregulation affects the systemic inflammatory state, which may be risky [143]. Simultaneously, the local inflammatory state caused by diseases changes the composition of the local microbiota [144], and the interaction between them affects the physiological state of the human body. Two theories were presented about “who comes first.” One states that microbes first change the microenvironment and accordingly lead to carcinogenesis, whereas the other states that changes in lesion sites attract bacteria. Therefore, long-term cohort studies are required to identify triggers and major factors. Perhaps the reality is a combination of both theories, as some bacteria, such as *F. nucleatum*, *P. gingivalis*, and *Streptococcus* species, show both tumor instigation and tumor tendency effects. Currently, microbiome descriptions mainly focus on bacteria, and further investigations on oral viruses are required. Beyond studying changes in microbial species and quantity, research should focus on the genomes and metabolites of microorganisms in the environment.

In addition to conventional surgery, chemotherapy, and radiotherapy, researchers are searching for other approaches to prevent OPMDS from progressing to OSCC and treat OSCC. Microbiota can be both a target for OSCC treatment and an adjunct to traditional therapies to enhance therapeutic effects or cut down on adverse effects. 

## Figures and Tables

**Table 1:** Summary of the role of microorganisms in the diagnosis, treatment, and prognosis of cancer.

Diagnosis and prognosis predicting markers	*Capnocytophaga gingivalis*, *Prevotella melaninogenica*, and *Streptococcus mitis* [90]
	*P. gingivalis* IgG and IL-6 [97]
	CXCL10, DIAPH1, NCLN, and MMP9 genes [98]
	*F. periodonticum*, *S. mitis*, and *P. pasteri* [24]
	*Fusobacterium periodonticum*, *Parvimonas micra*, *Streptococcus constellatus*, *Haemophilus influenza*, and *Filifactor alocis* [24]
	*Leptotrichia* [123,124]
	Tumoral MMP-9 [99]
Treatment targets	Tacrolimus [100,101]
	*Bifidobacterium*, *Streptococcus*, *Caulobacter*, and *Clostridium* spp. [116]
Affecting treatment outcome	Gammaproteobacteria convert gemcitabine into its inactive form by expressing the bacterial enzyme cytidine deaminase [126]
	*preTA*-encoding bacteria metabolizes 5-FU [127]
	Gut microbiota enhances chemotherapy-induced mechanical hyperalgesia [128]
	*Fusobacteria* species promote the differentiation of T cells [129]
	Antibiotic-mediated fungal reduction enhances the response to radiation and antibiotic-mediated bacteria reduction presents the opposite results [130]
	Palifermin [138]
	Lactococci strains relieve OM caused by 5-FU [141]

## References

[1] Warnakulasuriya S. (2009). Global epidemiology of oral and oropharyngeal cancer. Oral Oncol..

[2] Amit M., Yen T.C., Liao C.T., Chaturvedi P., Agarwal J.P., Kowalski L.P., Ebrahimi A., Clark J.R., Kreppel M., Zöller J. (2013). Improvement in survival of patients with oral cavity squamous cell carcinoma: An international collaborative study. Cancer.

[3] Sung H., Ferlay J., Siegel R.L., Laversanne M., Soerjomataram I., Jemal A., Bray F. (2021). Global Cancer Statistics 2020: GLOBOCAN Estimates of Incidence and Mortality Worldwide for 36 Cancers in 185 Countries. CA Cancer J. Clin..

[4] Hoes L., Dok R., Verstrepen K.J., Nuyts S. (2021). Ethanol-Induced Cell Damage Can Result in the Development of Oral Tumors. Cancers.

[5] Ford P.J., Rich A.M. (2021). Tobacco Use and Oral Health. Addiction.

[6] Wang W.-C., Chiu Y.-T., Wang Y.-Y., Lu S.-L., Chan L.-P., Lee C.-Y., Yang F.M., Yuan S.F., Lee C.-H. (2022). Effects of *DSM-5* Betel-Quid-Related Symptoms, Pathological Behaviors, and Use Disorder on Oral Squamous Cell Carcinoma Risk. Cancers.

[7] Healy C.M., Moran G.P. (2019). The microbiome and oral cancer: More questions than answers. Oral Oncol..

[8] Ganly I., Yang L., Giese R.A., Hao Y., Nossa C.W., Morris L.G., Rosenthal M., Migliacci J., Kelly D., Tseng W. (2019). Periodontal pathogens are a risk factor of oral cavity squamous cell carcinoma, independent of tobacco and alcohol and human papillomavirus. Int. J. Cancer.

[9] Karpiński T.M. (2019). Role of Oral Microbiota in Cancer Development. Microorganisms.

[10] Yao X., Smolka A.J. (2019). Gastric Parietal Cell Physiology and Helicobacter pylori–Induced Disease. Gastroenterology.

[11] Tierney B.T., Yang Z., Luber J.M., Beaudin M., Wibowo M.C., Baek C., Mehlenbacher E., Patel C.J., Kostic A.D. (2019). The Landscape of Genetic Content in the Gut and Oral Human Microbiome. Cell Host Microbe.

[12] Stasiewicz M., Karpiński T.M. (2022). The oral microbiota and its role in carcinogenesis. Semin. Cancer Biol..

[13] Iocca O., Sollecito T.P., Alawi F., Weinstein G.S., Newman J.G., De Virgilio A., Di Maio P., Spriano G., López S.P., Shanti R.M. (2020). Potentially malignant disorders of the oral cavity and oral dysplasia: A systematic review and meta-analysis of malignant transformation rate by subtype. Head Neck.

[14] Kumari P., Debta P., Dixit A. (2022). Oral Potentially Malignant Disorders: Etiology, Pathogenesis, and Transformation into Oral Cancer. Front. Pharmacol..

[15] Hashimoto K., Shimizu D., Ueda S., Miyabe S., Oh-Iwa I., Nagao T., Shimozato K., Nomoto S. (2022). Feasibility of oral microbiome profiles associated with oral squamous cell carcinoma. J. Oral Microbiol..

[16] Amer A., Galvin S., Healy C.M., Moran G.P. (2017). The Microbiome of Potentially Malignant Oral Leukoplakia Exhibits Enrichment for *Fusobacterium*, *Leptotrichia*, *Campylobacter*, and *Rothia* Species. Front. Microbiol..

[17] Pietrobon G., Tagliabue M., Stringa L.M., De Berardinis R., Chu F., Zocchi J., Carlotto E., Chiocca S., Ansarin M. (2021). Leukoplakia in the Oral Cavity and Oral Microbiota: A Comprehensive Review. Cancers.

[18] Gopinath D., Menon R.K., Wie C.C., Banerjee M., Panda S., Mandal D., Behera P.K., Roychoudhury S., Kheur S., Botelho M.G. (2020). Salivary bacterial shifts in oral leukoplakia resemble the dysbiotic oral cancer bacteriome. J. Oral Microbiol..

[19] Halboub E., Al-Ak’hali M.S., Alamir A.H., Homeida H.E., Baraniya D., Chen T., Al-Hebshi N.N. (2020). Tongue microbiome of smokeless tobacco users. BMC Microbiol..

[20] Vadovics M., Ho J., Igaz N., Alföldi R., Rakk D., Veres É., Szücs B., Horváth M., Tóth R., Szücs A. (2022). *Candida albicans* Enhances the Progression of Oral Squamous Cell Carcinoma In Vitro and In Vivo. mBio.

[21] Shang Q., Peng J., Zhou Y., Chen Q., Xu H. (2020). Association of Human Papillomavirus with Oral Lichen Planus and Oral Leukoplakia: A Meta-analysis. J. Évid. Based Dent. Pract..

[22] Khan M.M., Frustino J., Villa A., Nguyen B.-C., Woo S.-B., Johnson W.E., Varelas X., Kukuruzinska M., Monti S. (2023). Total RNA sequencing reveals gene expression and microbial alterations shared by oral pre-malignant lesions and cancer. Hum. Genom..

[23] Robledo-Sierra J., Ben-Amy D.P., Varoni E., Bavarian R., Simonsen J.L., Paster B.J., Wade W.G., Kerr R., Peterson D.E., Lau E.F. (2019). World Workshop on Oral Medicine VII: Targeting the oral microbiome Part 2: Current knowledge on malignant and potentially malignant oral disorders. Oral Dis..

[24] Yang C.-Y., Yeh Y.-M., Yu H.-Y., Chin C.-Y., Hsu C.-W., Liu H., Huang P.-J., Hu S.-N., Liao C.-T., Chang K.-P. (2018). Oral Microbiota Community Dynamics Associated with Oral Squamous Cell Carcinoma Staging. Front. Microbiol..

[25] Hsiao J.-R., Chang C.-C., Lee W.-T., Huang C.-C., Ou C.-Y., Tsai S.-T., Chen K.-C., Huang J.-S., Wong T.-Y., Lai Y.-H. (2018). The interplay between oral microbiome, lifestyle factors and genetic polymorphisms in the risk of oral squamous cell carcinoma. Carcinogens.

[26] Kavarthapu A., Gurumoorthy K. (2021). Linking chronic periodontitis and oral cancer: A review. Oral Oncol..

[27] Hayes R.B., Ahn J., Fan X., Peters B.A., Ma Y., Yang L., Agalliu I., Burk R.D., Ganly I., Purdue M.P. (2018). Association of Oral Microbiome with Risk for Incident Head and Neck Squamous Cell Cancer. JAMA Oncol..

[28] Li Z., Liu Y., Zhang L. (2022). Role of the microbiome in oral cancer occurrence, progression and therapy. Microb. Pathog..

[29] Szymonowicz K.A., Chen J. (2020). Biological and clinical aspects of HPV-related cancers. Cancer Biol. Med..

[30] Zebardast A., Yahyapour Y., Majidi M.S., Chehrazi M., Sadeghi F. (2021). Detection of Epstein-Barr virus encoded small RNA genes in oral squamous cell carcinoma and non-cancerous oral cavity samples. BMC Oral Health.

[31] Yang Y., Cai Q., Shu X.O., Steinwandel M.D., Blot W.J., Zheng W., Long J. (2019). Prospective study of oral microbiome and colorectal cancer risk in low-income and African American populations. Int. J. Cancer.

[32] Aslani N., Janbabaei G., Abastabar M., Meis J.F., Babaeian M., Khodavaisy S., Boekhout T., Badali H. (2018). Identification of uncommon oral yeasts from cancer patients by MALDI-TOF mass spectrometry. BMC Infect. Dis..

[33] Krogh P. (1990). The role of yeasts in oral cancer by means of endogenous nitrosation. Acta Odontol. Scand..

[34] Takeuchi H., Setoguchi T., Machigashira M., Kanbara K., Izumi Y. (2008). Hydrogen sulfide inhibits cell proliferation and induces cell cycle arrest via an elevated p21Cip1 level in Ca9-22 cells. J. Periodontal Res..

[35] Yaegaki K., Qian W., Murata T., Imai T., Sato T., Tanaka T., Kamoda T. (2008). Oral malodorous compound causes apoptosis and genomic DNA damage in human gingival fibroblasts. J. Periodontal Res..

[36] Sami A., Elimairi I., Stanton C., Ross R.P., Ryan C.A. (2020). The Role of the Microbiome in Oral Squamous Cell Carcinoma with Insight into the Microbiome–Treatment Axis. Int. J. Mol. Sci..

[37] Muto M., Hitomi Y., Ohtsu A., Shimada H., Kashiwase Y., Sasaki H., Yoshida S., Esumi H. (2000). Acetaldehyde production by non-pathogenic *Neisseria* in human oral microflora: Implications for carcinogenesis in upper aerodigestive tract. Int. J. Cancer.

[38] Blaser M.J. (2008). Understanding microbe-induced cancers. Cancer Prev. Res..

[39] Alnuaimi A.D., Ramdzan A.N., Wiesenfeld D., O’Brien-Simpson N.M., Kolev S.D., Reynolds E.C., McCullough M.J. (2016). *Candida* virulence and ethanol-derived acetaldehyde production in oral cancer and non-cancer subjects. Oral Dis..

[40] Smędra A., Berent J. (2023). The Influence of the Oral Microbiome on Oral Cancer: A Literature Review and a New Approach. Biomolecules.

[41] Balkwill F., Mantovani A. (2001). Inflammation and cancer: Back to Virchow?. Lancet.

[42] Coussens L.M., Werb Z. (2002). Inflammation and cancer. Nature.

[43] Yao Y., Shen X., Zhou M., Tang B. (2021). Periodontal Pathogens Promote Oral Squamous Cell Carcinoma by Regulating ATR and NLRP3 Inflammasome. Front. Oncol..

[44] Nieminen M.T., Listyarifah D., Hagström J., Haglund C., Grenier D., Nordström D., Uitto V.J., Hernandez M., Yucel-Lindberg T., Tervahartiala T. (2018). *Treponema denticola* chymotrypsin-like proteinase may contribute to orodigestive carcinogenesis through immunomodulation. Br. J. Cancer.

[45] Ren J., Han X., Lohner H., Hoyle R.G., Li J., Liang S., Wang H. (2023). *P. gingivalis* Infection Upregulates PD-L1 Expression on Dendritic Cells, Suppresses CD8^+^ T-cell Responses, and Aggravates Oral Cancer. Cancer Immunol. Res..

[46] Arjunan P., Meghil M.M., Pi W., Xu J., Lang L., El-Awady A., Sullivan W., Rajendran M., Rabelo M.S., Wang T. (2018). Oral Pathobiont Activates Anti-Apoptotic Pathway, Promoting both Immune Suppression and Oncogenic Cell Proliferation. Sci. Rep..

[47] Yuan X., Liu Y., Li G., Lan Z., Ma M., Li H., Kong J., Sun J., Hou G., Hou X. (2019). Blockade of Immune-Checkpoint B7-H4 and Lysine Demethylase 5B in Esophageal Squamous Cell Carcinoma Confers Protective Immunity against *P. gingivalis* Infection. Cancer Immunol. Res..

[48] Malinowski B., Węsierska A., Zalewska K., Sokołowska M.M., Bursiewicz W., Socha M., Ozorowski M., Pawlak-Osińska K., Wiciński M. (2019). The role of *Tannerella forsythia* and *Porphyromonas gingivalis* in pathogenesis of esophageal cancer. Infect. Agents Cancer.

[49] Gur C., Ibrahim Y., Isaacson B., Yamin R., Abed J., Gamliel M., Enk J., Bar-On Y., Stanietsky-Kaynan N., Coppenhagen-Glazer S. (2015). Binding of the Fap2 Protein of *Fusobacterium nucleatum* to Human Inhibitory Receptor TIGIT Protects Tumors from Immune Cell Attack. Immunity.

[50] Chen T., Li Q., Wu J., Wu Y., Peng W., Li H., Wang J., Tang X., Peng Y., Fu X. (2018). *Fusobacterium nucleatum* promotes M2 polarization of macrophages in the microenvironment of colorectal tumours via a TLR4-dependent mechanism. Cancer Immunol. Immunother. CII.

[51] Sun J., Tang Q., Yu S., Xie M., Zheng W., Chen G., Yin Y., Huang X., Wo K., Lei H. (2023). *F. nucleatum* facilitates oral squamous cell carcinoma progression via GLUT1-driven lactate production. EBioMedicine.

[52] Huang S.-M., McCance D.J. (2002). Down Regulation of the Interleukin-8 Promoter by Human Papillomavirus Type 16 E6 and E7 through Effects on CREB Binding Protein/p300 and P/CAF. J. Virol..

[53] Chang C., Wang H., Liu J., Pan C., Zhang D., Li X., Pan Y. (2019). *Porphyromonas gingivalis* Infection Promoted the Proliferation of Oral Squamous Cell Carcinoma Cells through the miR-21/PDCD4/AP-1 Negative Signaling Pathway. ACS Infect. Dis..

[54] Gao S., Liu Y., Duan X., Liu K., Mohammed M., Gu Z., Ren J., Yakoumatos L., Yuan X., Lu L. (2021). *Porphyromonas gingivalis* infection exacerbates oesophageal cancer and promotes resistance to neoadjuvant chemotherapy. Br. J. Cancer.

[55] Zhou Y., Sztukowska M., Wang Q., Inaba H., Potempa J., Scott D.A., Wang H., Lamont R.J. (2015). Noncanonical Activation of β-Catenin by *Porphyromonas gingivalis*. Infect. Immun..

[56] Geng F., Zhang Y., Lu Z., Zhang S., Pan Y. (2020). *Fusobacterium nucleatum* Caused DNA Damage and Promoted Cell Proliferation by the Ku70/p53 Pathway in Oral Cancer Cells. DNA Cell Biol..

[57] Ding N., Cheng Y., Liu H., Wu Y., Weng Y., Cui H., Cheng C., Zhang W., Cui Y. (2023). *Fusobacterium nucleatum* Infection Induces Malignant Proliferation of Esophageal Squamous Cell Carcinoma Cell by Putrescine Production. Microbiol. Spectr..

[58] Wang Y., Liu S., Li B., Jiang Y., Zhou X., Chen J., Li M., Ren B., Peng X., Zhou X. (2019). *Staphylococcus aureus* induces COX-2-dependent proliferation and malignant transformation in oral keratinocytes. J. Oral Microbiol..

[59] Lee J., Roberts J.S., Atanasova K.R., Chowdhury N., Yilmaz Ö. (2018). A novel kinase function of a nucleoside-diphosphate-kinase homologue in *Porphyromonas gingivalis* is critical in subversion of host cell apoptosis by targeting heat-shock protein 27. Cell. Microbiol..

[60] Mao S., Park Y., Hasegawa Y., Tribble G.D., James C.E., Handfield M., Stavropoulos M.F., Yilmaz Ö., Lamont R.J. (2007). Intrinsic apoptotic pathways of gingival epithelial cells modulated by *Porphyromonas gingivalis*. Cell. Microbiol..

[61] Yao L., Jermanus C., Barbetta B., Choi C., Verbeke P., Ojcius D.M., Yilmaz Ö. (2010). *Porphyromonas gingivalis* infection sequesters pro-apoptotic Bad through Akt in primary gingival epithelial cells. Mol. Oral Microbiol..

[62] Harrandah A.M., Chukkapalli S.S., Bhattacharyya I., Progulske-Fox A., Chan E.K.L. (2020). Fusobacteria modulate oral carcinogenesis and promote cancer progression. J. Oral Microbiol..

[63] Peng R.T., Sun Y., Zhou X.D., Liu S.Y., Han Q., Cheng L., Peng X. (2022). *Treponema denticola* Promotes OSCC Development via the TGF-β Signaling Pathway. J. Dent. Res..

[64] Song Y., Wu X., Xu Y., Zhu J., Li J., Zou Z., Chen L., Zhang B., Hua C., Rui H. (2020). HPV E7 inhibits cell pyroptosis by promoting TRIM21-mediated degradation and ubiquitination of the IFI16 inflammasome. Int. J. Biol. Sci..

[65] Karlsson M.C., Gonzalez S.F., Welin J., Fuxe J. (2017). Epithelial-mesenchymal transition in cancer metastasis through the lymphatic system. Mol. Oncol..

[66] Guo Z.-C., Jing S.-L., Jumatai S., Gong Z.-C. (2023). *Porphyromonas gingivalis* promotes the progression of oral squamous cell carcinoma by activating the neutrophil chemotaxis in the tumour microenvironment. Cancer Immunol. Immunother. CII.

[67] Xu M., Yamada M., Li M., Liu H., Chen S.G., Han Y.W. (2007). FadA from *Fusobacterium nucleatum* Utilizes both Secreted and Nonsecreted Forms for Functional Oligomerization for Attachment and Invasion of Host Cells. J. Biol. Chem..

[68] Eun Y.-G., Lee J.-W., Kim S.W., Hyun D.-W., Bae J.-W., Lee Y.C. (2021). Oral microbiome associated with lymph node metastasis in oral squamous cell carcinoma. Sci. Rep..

[69] Neuzillet C., Marchais M., Vacher S., Hilmi M., Schnitzler A., Meseure D., Leclere R., Lecerf C., Dubot C., Jeannot E. (2021). Prognostic value of intratumoral *Fusobacterium nucleatum* and association with immune-related gene expression in oral squamous cell carcinoma patients. Sci. Rep..

[70] Zhang S., Li C., Liu J., Geng F., Shi X., Li Q., Lu Z., Pan Y. (2020). *Fusobacterium nucleatum* promotes epithelial-mesenchymal transiton through regulation of the lncRNA MIR4435-2HG/miR-296-5p/Akt2/SNAI1 signaling pathway. FEBS J..

[71] Voronov E., Shouval D.S., Krelin Y., Cagnano E., Benharroch D., Iwakura Y., Dinarello C.A., Apte R.N. (2003). IL-1 is required for tumor invasiveness and angiogenesis. Proc. Natl. Acad. Sci. USA.

[72] Inaba H., Amano A., Lamont R.J., Murakami Y. (2015). Involvement of protease-activated receptor 4 in over-expression of matrix metalloproteinase 9 induced by *Porphyromonas gingivalis*. Med. Microbiol. Immunol..

[73] Broccolo F., Ciccarese G., Rossi A., Anselmi L., Drago F., Toniolo A. (2018). Human papillomavirus (HPV) and Epstein-Barr virus (EBV) in keratinizing versus non-keratinizing squamous cell carcinoma of the oropharynx. Infect. Agents Cancer.

[74] Ho J., Yang X., Nikou S.-A., Kichik N., Donkin A., Ponde N.O., Richardson J.P., Gratacap R.L., Archambault L.S., Zwirner C.P. (2019). Candidalysin activates innate epithelial immune responses via epidermal growth factor receptor. Nat. Commun..

[75] Listyarifah D., Nieminen M.T., Mäkinen L.K., Haglund C., Grenier D., Häyry V., Nordström D., Hernandez M., Yucel-Lindberg T., Tervahartiala T. (2018). *Treponema denticola* chymotrypsin-like proteinase is present in early-stage mobile tongue squamous cell carcinoma and related to the clinicopathological features. J. Oral Pathol. Med. Off. Publ. Int. Assoc. Oral Pathol. Am. Acad. Oral Pathol..

[76] Kamarajan P., Ateia I., Shin J.M., Fenno J.C., Le C., Zhan L., Chang A., Darveau R., Kapila Y.L. (2020). Periodontal pathogens promote cancer aggressivity via TLR/MyD88 triggered activation of Integrin/FAK signaling that is therapeutically reversible by a probiotic bacteriocin. PLoS Pathog..

[77] Wang Q., Song R., Zhao C., Liu H., Yang Y., Gu S., Feng D., He J. (2019). HPV16 E6 promotes cervical cancer cell migration and invasion by downregulation of NHERF1. Int. J. Cancer.

[78] Madhura M.G., Rao R.S., Patil S., Fageeh H.N., Alhazmi A., Awan K.H. (2020). Advanced diagnostic aids for oral cancer. Disease-a-Month.

[79] Frank D.N., Qiu Y., Cao Y., Zhang S., Lu L., Kofonow J.M., Robertson C.E., Liu Y., Wang H., Levens C.L. (2022). A dysbiotic microbiome promotes head and neck squamous cell carcinoma. Oncogene.

[80] Su S.-C., Chang L.-C., Huang H.-D., Peng C.-Y., Chuang C.-Y., Chen Y.-T., Lu M.-Y., Chiu Y.-W., Chen P.-Y., Yang S.-F. (2021). Oral microbial dysbiosis and its performance in predicting oral cancer. Carcinogenesis.

[81] Arthur R.A., Dos Santos Bezerra R., Ximenez J.P.B., Merlin B.L., de Andrade Morraye R., Neto J.V., Fava N.M.N., Figueiredo D.L.A., de Biagi C.A.O., Montibeller M.J. (2021). Microbiome and oral squamous cell carcinoma: A possible interplay on iron metabolism and its impact on tumor microenvironment. Braz. J. Microbiol..

[82] Radaic A., Kamarajan P., Cho A., Wang S., Hung G.-C., Najarzadegan F., Wong D.T., Ton-That H., Wang C.-Y., Kapila Y.L. (2023). Biological biomarkers of oral cancer. Periodontology 2000.

[83] Wang S., Yang M., Li R., Bai J. (2023). Current advances in noninvasive methods for the diagnosis of oral squamous cell carcinoma: A review. Eur. J. Med. Res..

[84] Nejman D., Livyatan I., Fuks G., Gavert N., Zwang Y., Geller L.T., Rotter-Maskowitz A., Weiser R., Mallel G., Gigi E. (2020). The human tumor microbiome is composed of tumor type–specific intracellular bacteria. Science.

[85] Singh R.P., Kumari N., Gupta S., Jaiswal R., Mehrotra D., Singh S., Mukherjee S., Kumar R. (2023). Intratumoral Microbiota Changes with Tumor Stage and Influences the Immune Signature of Oral Squamous Cell Carcinoma. Microbiol. Spectr..

[86] Hooper S.J., Crean S.J., Lewis M.A.O., Spratt D.A., Wade W.G., Wilson M.J. (2006). Viable Bacteria Present within Oral Squamous Cell Carcinoma Tissue. J. Clin. Microbiol..

[87] Kumar P., Gupta S., Das B.C. (2023). Saliva as a potential non-invasive liquid biopsy for early and easy diagnosis/prognosis of head and neck cancer. Transl. Oncol..

[88] Heng W., Wang W., Dai T., Jiang P., Lu Y., Li R., Zhang M., Xie R., Zhou Y., Zhao M. (2022). Oral Bacteriome and Mycobiome across Stages of Oral Carcinogenesis. Microbiol. Spectr..

[89] Lee W.-H., Chen H.-M., Yang S.-F., Liang C., Peng C.-Y., Lin F.-M., Tsai L.-L., Wu B.-C., Hsin C.-H., Chuang C.-Y. (2017). Bacterial alterations in salivary microbiota and their association in oral cancer. Sci. Rep..

[90] Mager D.L., Haffajee A.D., Devlin P.M., Norris C.M., Posner M.R., Goodson J.M. (2005). The salivary microbiota as a diagnostic indicator of oral cancer: A descriptive, non-randomized study of cancer-free and oral squamous cell carcinoma subjects. J. Transl. Med..

[91] Zhou X., Hao Y., Peng X., Li B., Han Q., Ren B., Li M., Li L., Li Y., Cheng G. (2021). The Clinical Potential of Oral Microbiota as a Screening Tool for Oral Squamous Cell Carcinomas. Front. Cell. Infect. Microbiol..

[92] Schmidt B.L., Kuczynski J., Bhattacharya A., Huey B., Corby P.M., Queiroz E.L.S., Nightingale K., Kerr A.R., DeLacure M.D., Veeramachaneni R. (2014). Changes in Abundance of Oral Microbiota Associated with Oral Cancer. PLoS ONE.

[93] Al-Hebshi N.N., Nasher A.T., Maryoud M.Y., Homeida H.E., Chen T., Idris A.M., Johnson N.W. (2017). Inflammatory bacteriome featuring *Fusobacterium nucleatum* and *Pseudomonas aeruginosa* identified in association with oral squamous cell carcinoma. Sci. Rep..

[94] Zhao Q., Yang T., Yan Y., Zhang Y., Li Z., Wang Y., Yang J., Xia Y., Xiao H., Han H. (2020). Alterations of Oral Microbiota in Chinese Patients with Esophageal Cancer. Front. Cell. Infect. Microbiol..

[95] Peng Q.-S., Cheng Y.-N., Zhang W.-B., Fan H., Mao Q.-H., Xu P. (2020). circRNA_0000140 suppresses oral squamous cell carcinoma growth and metastasis by targeting miR-31 to inhibit Hippo signaling pathway. Cell Death Dis..

[96] Shen X., Zhang B., Hu X., Li J., Wu M., Yan C., Yang Y., Li Y. (2022). *Neisseria sicca* and *Corynebacterium matruchotii* inhibited oral squamous cell carcinomas by regulating genome stability. Bioengineered.

[97] Park D.-G., Woo B.H., Lee B.-J., Yoon S., Cho Y., Kim Y.-D., Park H.R., Song J.M. (2019). Serum Levels of Interleukin-6 and Titers of Antibodies against *Porphyromonas gingivalis* Could Be Potential Biomarkers for the Diagnosis of Oral Squamous Cell Carcinoma. Int. J. Mol. Sci..

[98] Moghimi M., Bakhtiari R., Mehrabadi J.F., Jamshidi N., Jamshidi N., Siyadatpanah A., Mitsuwan W., Nissapatorn V. (2020). Interaction of human oral cancer and the expression of virulence genes of dental pathogenic bacteria. Microb. Pathog..

[99] Kylmä A.K., Sorsa T., Jouhi L., Mustonen H.K., Mohamed H., Randén-Brady R., Mäkitie A., Atula T., Hagström J., Haglund C. (2022). Prognostic Role of *Porphyromonas gingivalis* Gingipain Rgp and Matrix Metalloproteinase 9 in Oropharyngeal Squamous Cell Carcinoma. Anticancer Res..

[100] Utz S., Suter V.G.A., Cazzaniga S., Borradori L., Feldmeyer L. (2022). Outcome and long-term treatment protocol for topical tacrolimus in oral lichen planus. J. Eur. Acad. Dermatol. Venereol..

[101] Polizzi A., Santonocito S., Lo Giudice A., Alibrandi A., De Pasquale R., Isola G. (2023). Analysis of the response to two pharmacological protocols in patients with oral lichen planus: A randomized clinical trial. Oral Dis..

[102] Mok C.C., Tong K.H., To C.H., Siu Y.P., Au T.C. (2005). Tacrolimus for induction therapy of diffuse proliferative lupus nephritis: An open-labeled pilot study. Kidney Int..

[103] Li Y., Wang Y., Li J., Ling Z., Chen W., Zhang L., Hu Q., Wu T., Cheng B., Wang Y. (2021). Tacrolimus inhibits oral carcinogenesis through cell cycle control. Biomed. Pharmacother..

[104] Zhang Z., Liu L., Tang H., Jiao W., Zeng S., Xu Y., Zhang Q., Sun Z., Mukherjee A., Zhang X. (2018). Immunosuppressive effect of the gut microbiome altered by high-dose tacrolimus in mice. Am. J. Transplant..

[105] Sun S.L., Liu J.J., Zhong B., Wang J.K., Jin X., Xu H., Yin F.Y., Liu T.N., Chen Q.M., Zeng X. (2019). Topical calcineurin inhibitors in the treatment of oral lichen planus: A systematic review and meta-analysis. Br. J. Dermatol..

[106] Cheng J.Y., Li F.-Y., Ko C.J., Colegio O.R. (2018). Cutaneous Squamous Cell Carcinomas in Solid Organ Transplant Recipients Compared with Immunocompetent Patients. JAMA Dermatol..

[107] Zaalberg A., Tuchayi S.M., Ameri A.H., Ngo K.H., Cunningham T.J., Eliane J.-P., Livneh M., Horn T.D., Rosman I.S., Musiek A. (2019). Chronic Inflammation Promotes Skin Carcinogenesis in Cancer-Prone Discoid Lupus Erythematosus. J. Investig. Dermatol..

[108] Veitch M., Beaumont K., Pouwer R., Chew H.Y., Frazer I.H., Soyer H.P., Campbell S., Dymock B.W., Harvey A., Cock T.-A. (2023). Local blockade of tacrolimus promotes T-cell-mediated tumor regression in systemically immunosuppressed hosts. J. Immunother. Cancer.

[109] Su Z., Hu J., Cheng B., Tao X. (2022). Efficacy and safety of topical administration of tacrolimus in oral lichen planus: An updated systematic review and meta-analysis of randomized controlled trials. J. Oral Pathol. Med. Off. Publ. Int. Assoc. Oral Pathol. Am. Acad. Oral Pathol..

[110] Mäkinen A.I., Pappalardo V.Y., Buijs M.J., Brandt B.W., Mäkitie A.A., Meurman J.H., Zaura E. (2023). Salivary microbiome profiles of oral cancer patients analyzed before and after treatment. Microbiome.

[111] Diwan P., Nirwan M., Bahuguna M., Kumari S.P., Wahlang J., Gupta R.K. (2023). Evaluating Alterations of the Oral Microbiome and Its Link to Oral Cancer among Betel Quid Chewers: Prospecting Reversal through Probiotic Intervention. Pathogens.

[112] Zhou S., Gravekamp C., Bermudes D., Liu K. (2018). Tumour-targeting bacteria engineered to fight cancer. Nat. Rev. Cancer.

[113] Xiong S., Qi Z., Ni J., Zhong J., Cao L., Yang K. (2020). Attenuated *Salmonella typhimurium*-mediated tumour targeting imaging based on peptides. Biomater. Sci..

[114] Yu X., Lin C., Yu J., Qi Q., Wang Q. (2020). Bioengineered *Escherichia coli* Nissle 1917 for tumour-targeting therapy. Microb. Biotechnol..

[115] Li Q., Li Y., Wang Y., Xu L., Guo Y., Wang Y., Wang L., Guo C. (2021). Oral administration of *Bifidobacterium breve* promotes antitumor efficacy via dendritic cells-derived interleukin 12. OncoImmunology.

[116] Liu S., Xu X., Zeng X., Li L., Chen Q., Li J. (2014). Tumor-targeting bacterial therapy: A potential treatment for oral cancer (Review). Oncol. Lett..

[117] Wedge M.-E., Jennings V.A., Crupi M.J.F., Poutou J., Jamieson T., Pelin A., Pugliese G., de Souza C.T., Petryk J., Laight B.J. (2022). Virally programmed extracellular vesicles sensitize cancer cells to oncolytic virus and small molecule therapy. Nat. Commun..

[118] Dróżdż M., Makuch S., Cieniuch G., Woźniak M., Ziółkowski P. (2020). Obligate and facultative anaerobic bacteria in targeted cancer therapy: Current strategies and clinical applications. Life Sci..

[119] Guo Y., Song M., Liu X., Chen Y., Xun Z., Sun Y., Tan W., He J., Zheng J.H. (2022). Photodynamic therapy-improved oncolytic bacterial immunotherapy with FAP-encoding *S. typhimurium*. J. Control. Release.

[120] Lou X., Chen Z., He Z., Sun M., Sun J. (2021). Bacteria-Mediated Synergistic Cancer Therapy: Small Microbiome Has a Big Hope. Nano-Micro Lett..

[121] Panebianco C., Andriulli A., Pazienza V. (2018). Pharmacomicrobiomics: Exploiting the drug-microbiota interactions in anticancer therapies. Microbiome.

[122] Medeiros M.C., The S., Bellile E., Russo N., Schmitd L., Danella E., Singh P., Banerjee R., Bassis C., Murphy G.R. (2023). Salivary microbiome changes distinguish response to chemoradiotherapy in patients with oral cancer. Microbiome.

[123] Hamada M., Inaba H., Nishiyama K., Yoshida S., Yura Y., Matsumoto-Nakano M., Uzawa N. (2023). Potential Role of the Intratumoral Microbiota in Prognosis of Head and Neck Cancer. Int. J. Mol. Sci..

[124] Lyu W.-N., Lin M.-C., Shen C.-Y., Chen L.-H., Lee Y.-H., Chen S.-K., Lai L.-C., Chuang E.Y., Lou P.-J., Tsai M.-H. (2023). An Oral Microbial Biomarker for Early Detection of Recurrence of Oral Squamous Cell Carcinoma. ACS Infect. Dis..

[125] Lehouritis P., Cummins J., Stanton M., Murphy C.T., McCarthy F.O., Reid G., Urbaniak C., Byrne W.L., Tangney M. (2015). Local bacteria affect the efficacy of chemotherapeutic drugs. Sci. Rep..

[126] Geller L.T., Barzily-Rokni M., Danino T., Jonas O.H., Shental N., Nejman D., Gavert N., Zwang Y., Cooper Z.A., Shee K. (2017). Potential role of intratumor bacteria in mediating tumor resistance to the chemotherapeutic drug gemcitabine. Science.

[127] Spanogiannopoulos P., Kyaw T.S., Guthrie B.G.H., Bradley P.H., Lee J.V., Melamed J., Malig Y.N.A., Lam K.N., Gempis D., Sandy M. (2022). Host and gut bacteria share metabolic pathways for anti-cancer drug metabolism. Nat. Microbiol..

[128] Shen S., Lim G., You Z., Ding W., Huang P., Ran C., Doheny J., Caravan P., Tate S., Hu K. (2017). Gut microbiota is critical for the induction of chemotherapy-induced pain. Nat. Neurosci..

[129] Cremonesi E., Governa V., Garzon J.F.G., Mele V., Amicarella F., Muraro M.G., Trella E., Galati-Fournier V., Oertli D., Däster S.R. (2018). Gut microbiota modulate T cell trafficking into human colorectal cancer. Gut.

[130] Shiao S.L., Kershaw K.M., Limon J.J., You S., Yoon J., Ko E.Y., Guarnerio J., Potdar A.A., McGovern D.P.B., Bose S. (2021). Commensal bacteria and fungi differentially regulate tumor responses to radiation therapy. Cancer Cell.

[131] Li Z., Fu R., Huang X., Wen X., Zhang L. (2023). Oral microbiota may affect osteoradionecrosis following radiotherapy for head and neck cancer. J. Transl. Med..

[132] McDonnell A.M., Lenz K.L. (2007). Palifermin: Role in the Prevention of Chemotherapy- and Radiation-Induced Mucositis. Ann. Pharmacother..

[133] von Bültzingslöwen I., Adlerberth I., Wold A.E., Dahlén G., Jontell M. (2003). Oral and intestinal microflora in 5-fluorouracil treated rats, translocation to cervical and mesenteric lymph nodes and effects of probiotic bacteria. Oral Microbiol. Immunol..

[134] Hong B.-Y., Sobue T., Choquette L., Dupuy A.K., Thompson A., Burleson J.A., Salner A.L., Schauer P.K., Joshi P., Fox E. (2019). Chemotherapy-induced oral mucositis is associated with detrimental bacterial dysbiosis. Microbiome.

[135] Fernández Forné Á., García Anaya M.J., Segado Guillot S.J., Plaza Andrade I., de la Peña Fernández L., Lorca Ocón M.J., Lupiáñez Pérez Y., Queipo-Ortuño M.I., Gómez-Millán J. (2023). Influence of the microbiome on radiotherapy-induced oral mucositis and its management: A comprehensive review. Oral Oncol..

[136] Fallah M., Amin N., Moghaddasian M.H., Jafarnejad S. (2023). Probiotics for the Management of Oral Mucositis: An Interpretive Review of Current Evidence. Adv. Pharm. Bull..

[137] Vadhan-Raj S., Goldberg J.D., Perales M.A., Berger D.P., van den Brink M.R. (2013). Clinical applications of palifermin: Amelioration of oral mucositis and other potential indications. J. Cell. Mol. Med..

[138] Coutsouvelis J., Corallo C., Spencer A., Avery S., Dooley M., Kirkpatrick C.M. (2022). A meta-analysis of palifermin efficacy for the management of oral mucositis in patients with solid tumours and haematological malignancy. Crit. Rev. Oncol. Hematol..

[139] Bohn B., Chalupova M., Staley C., Holtan S., Maakaron J., Bachanova V., El Jurdi N. (2023). Temporal variation in oral microbiome composition of patients undergoing autologous hematopoietic cell transplantation with keratinocyte growth factor. BMC Microbiol..

[140] Patel P., Robinson P.D., Baggott C., Gibson P., Ljungman G., Massey N., Ottaviani G., Phillips R., Revon-Rivière G., Treister N. (2021). Clinical practice guideline for the prevention of oral and oropharyngeal mucositis in pediatric cancer and hematopoietic stem cell transplant patients: 2021 update. Eur. J. Cancer.

[141] Carvalho R., Vaz A., Pereira F.L., Dorella F., Aguiar E., Chatel J.-M., Bermudez L., Langella P., Fernandes G., Figueiredo H. (2018). Gut microbiome modulation during treatment of mucositis with the dairy bacterium *Lactococcus lactis* and recombinant strain secreting human antimicrobial PAP. Sci. Rep..

[142] Vandeputte D., Kathagen G., D’hoe K., Vieira-Silva S., Valles-Colomer M., Sabino J., Wang J., Tito R.Y., De Commer L., Darzi Y. (2017). Quantitative microbiome profiling links gut community variation to microbial load. Nature.

[143] Kleinstein S.E., Nelson K.E., Freire M. (2020). Inflammatory Networks Linking Oral Microbiome with Systemic Health and Disease. J. Dent. Res..

[144] Graves D.T., Corrêa J.D., Silva T.A. (2019). The Oral Microbiota Is Modified by Systemic Diseases. J. Dent. Res..

